# Commentary: Accelerating the science and practice of psychology beyond WEIRD biases: enriching the landscape through Asian psychology

**DOI:** 10.3389/fpsyg.2024.1260468

**Published:** 2024-09-18

**Authors:** Christopher Kam

**Affiliations:** Psychology Department, Adams State University, Alamosa, CO, United States

**Keywords:** Asian philosophy, dialectics, WEIRD and non-WEIRD societies, ego development, multiculturalism

## Introduction

Wong and Cowden (2022) write an insightful article outlining how Asian psychology (i.e., non-duality/dialectical interaction, Wu-Wei, and Zhong Yong) can broaden traditional psychology's bias toward a WEIRD (Western, Educated, Industrialized, Rich, and Democratic) orientation. They offer both theoretical and concrete examples for this. They write that WEIRD and non-WEIRD scholars should “consider forming collaborative partnerships with well-established international researchers who could provide expertise in developing and implementing systematic programs of research (Cowden et al., 2023)” (p. 5). They also write that these efforts can be helped by “integrative models that synthesize disparate theoretical orientations” (p. 5).

In this paper, I will give an opinion on how this international collaboration could begin with WEIRD and non-WEIRD scholars with an example of this cross-cultural conceptualization the way that Wong and Cowden (2022) propose. Here, I will show how scholars from the West who adopt a post-conventional mindset on adult development can collaborate with Asian scholars who work with assumptions of non-duality and dialectical interaction. I will then make the case for how Asian philosophy can, in turn, benefit from Western empirical research on the distinction and mutual interaction between conscious and unconscious processes.

## Example of WEIRD and non-WEIRD integration and collaboration

Certain subfields of adult developmental psychology conceptualize pre-conventional, conventional, and post-conventional levels of adult ego development (Cook-Greuter, 1999; Kohlberg, 1984). In the pre-conventional phase, people are predominantly living by premises that are self-serving. In the conventional phase, individuals adopt societal norms for life outlooks and on human nature. In the post-conventional phase, however, people transcend their society's cliches and norms for conceptualizing themselves, others, and society. This post-conventional outlook adopts assumptions of human nature that are no longer rigid, static, and unidimensional (Cook-Greuter, 2004; Loevinger, 1976). Post-conventional outlooks on life become open to paradox and the tension of holding opposites simultaneously. Here, there is no longer an obsession with rationally solving their coherence. Paradox with simultaneous opposites is accepted as a way of life along with comfort for ambiguity (Hy and Loevinger, 1996; Kam and Bellehumeur, 2020; Vincent et al., 2015). When this happens, a person is able to transcend the system of their society's thinking and flexibly adopt other systems of thinking interchangeably and/or simultaneously (Kegan, 1979; Kegan et al., 1998). By its very nature, post-conventional thinking in adult developmental psychology transcends WEIRD assumptions that emphasize linear coherence and structural consistency. This allows fertile mental soil for Asian non-duality and dialectical thinking. Wong and Cowden (2022) note that “Non-duality looks at everything in terms of wholeness based on Yin-Yang integration of independent but interactive opposing dimensions” (p. 2). This type of non-duality is more in line with “naïve dialectics” where everything is connected as a whole, as opposed to Western/scientific dialectics where the emphasis is rationally grasping how a paradox does not defy the law of non-contradiction (Peng and Nisbett, 1999). This is consistent with Loevinger (1976)'s conception of how higher stages of adult maturation, which Cook-Greuter (1999) sees as postconventional, transcend either-or thinking and is comfortable with the paradoxical tension of opposites mutually coexisting (Hy and Loevinger, 1996; Kam, 2023; Kam and Bellehumeur, 2021). By transcending their own WEIRD conventions, post-conventional adult maturity researchers in the spirit of Loevinger (1976), Cook-Greuter (1999), and Kegan et al. (1998), are conceptually open to theorizing about human nature and development in line with “helping people become aware of Yin-Yang dialectics and figure out more adaptive ways to embrace and transcend opposite forces (Wong, 2016)” (p. 2).

One possible hypothesis is that although both Western/scientific dialectics and naïve dialects may be accessible to both WEIRD and non-WEIRD populations when reaching the post-conventional stages of ego development, both populations may have general inclinations for one and not the other. For example, WEIRD post-conventional individuals may be more inclined to use Western/scientific dialectics to address issues involving the physical world (e.g., merging insights of Newtonian physics and quantum mechanics to solve cutting edge physics problems). In contrast, non-WEIRD post-conventional individuals may be more inclined to use naïve dialectics to address relational issues involving the self's connection with others (e.g., how to remain humble and authoritative at the same time, or how to be emotionally mature and have childlike wonder at the same time). These hypotheses invite multicultural methodologies for exploration.

## Cultivating conscious and unconscious non-duality

As there is basis for differentiating between conscious and unconscious complexity in mental life (Kam and Bellehumeur, 2021, 2022). In terms of tackling both conscious and unconscious dimensions of Western/scientific dialectics as well as naïve dialectics, methodological cross pollination is invited. For example, while post-conventional Western scholars can benefit from Asian conceptualizations of human nature such as non-duality, Asian scholars, in turn, can also benefit from WEIRD's established empirical framework of conscious and unconscious processes. There have been decades of accumulated quantitative research on how conscious and unconscious processes are distinct yet mutually interacting (Barbosa et al., 2017; Baumeister et al., 2017; Bettiga et al., 2017; Kam, 2022; Kiefer and Martens, 2010). For some time, researchers have noted how both conscious and unconscious processes solve complex problems better in collaboration than alone (Kam, 2023). Conscious mental activity is smaller in capacity but better at sequential reasoning while unconscious mental activity has more breadth, is more intuitive, and gravitates toward divergence and creativity (Dijksterhuis and Nordgren, 2006; Lewicki et al., 1992; Nordgren, 2011; Vieira et al., 2017). This cross pollination between WEIRD and non-WEIRD frameworks can happen at both the research and clinical level, expanding the conversation on cultivating both conscious and unconscious layers of non-duality, since doing so is beneficial to mature happiness (Wong and Bowers, 2018).

For the research level, both quantitative and qualitative research designs can explore how conscious and unconscious dimensions of dialectical complexity can reinforce each other (or get in the way of doing so). Experimental designs can contrast the pre-post results of different forms of dialectical complexity in conscious and unconscious ways with instruments that measure both (Kam, 2023) in experimental groups who undergo therapy with Asian philosophy compared to control groups. In tandem with this, qualitative studies can do semi-structured interviews with Interpretative Phenomenological Analysis (Smith et al., 2009) to explore how Western/scientific as well as naïve dialectics are experienced both consciously and unconsciously in individuals who have been experientially immersed in it for decades.

On the clinical level, the content of Asian dialectics can be explored both with interventions that focus on more conscious processes like Cognitive Behavioral Therapy as well as interventions that focus more on the unconscious like Jungian Psychoanalysis. For example, clients may experience that a wound from childhood trauma can lock an individual in either-or thinking (e.g., “I'm either strong or I'm weak”). Non-duality in this context may help the client explore the multidimensionality of one's humanness that has many sides in the diamond of one's personality. This can include formidable strengths in certain dimensions of oneself coexisting with the vulnerable side of the soul that's tender and hurtable, which ends up affirming one's humanness rather than denying it. This is in contrast to a 2-dimensional either-or rigid dualism that fails to transcend these seemingly opposite traits. More cognitive based therapies that focus on conscious processing can focus on psychoeducation and verbal processing of this 3-dimensionalizing content through talk therapy. More psychoanalytic therapies that focus on unconscious processing, such as dreamwork, can focus on more artistic, non-linear, and non-verbal means of processing the paradox and tension of opposites mysteriously coexisting.

Since research shows that ego development can transform from conventional to post-conventional levels through accommodative processing (Lilgendahl et al., 2013), there is opportunity to research how this can occur differently in WEIRD compared to non-WEIRD Asian contexts. Vincent et al. (2015) write:

“In order to promote the transition from conventional to post-conventional consciousness, developmental programs would presumably need to include experiences that expose participants to the fundamental paradoxes in human nature, confront them with ambiguous challenges and invite them to face their discomfort with this, as well as focusing their attention on their own mental habits and biases” (p. 241).

Since studies show that ego development toward post-conventional levels is facilitated by processing one's journey in a supportive community (Daniels et al., 2018; Kam and Bellehumeur, 2022), researchers can study how the transition from conventional to post-conventional dialectics can differ when confronting “fundamental paradoxes” and “ambiguous challenges” in WEIRD and non-WEIRD environments and discover advantages and shortcomings from each context.

## Discussion

This article explored a proposal by Wong and Cowden (2022) for WEIRD and non-WEIRD scholars to collaborate in a more concrete manner. An example was given to their proposal to conceptualize human nature and its flourishing in a more internationally rich manner. Specifically, post-conventional scholars on adult development and maturity have much opportunity to learn from Asian scholars on areas such as non-duality. In turn, Asian scholars can benefit from WEIRD research on conscious and unconscious processes and their mutual interaction. The result would be international enrichment on both research and clinical platforms, as transnational collaboration in human nature seems like a promising trend to tackle ongoing problems for humanity (Bellehumeur et al., 2022). This is not the only way to do this nonetheless. The hope is for attempts like these at concretizing WEIRD and non-WEIRD collaboration to excite the imagination of more scholars to dream and brainstorm ways Asian philosophy and Western understandings of human nature can multidimensionalize our understanding of maturation in a transcultural manner.
